# A Survey of Single‐Use and Reusable Surgical and Anaesthetic Devices in Victorian Public Health Services

**DOI:** 10.1111/ans.70500

**Published:** 2026-01-23

**Authors:** Elizabeth Peiwei Hu, Alexander Dillon, Forbes McGain, Jessica Davies

**Affiliations:** ^1^ Department of Anaesthesia Austin Health Heidelberg Australia; ^2^ Departments of Anaesthesia and Intensive Care Western Health Footscray Australia; ^3^ Department of Critical Care The University of Melbourne Melbourne Australia

## Abstract

**Objectives:**

To understand the adoption of reusable surgical and anaesthetic devices across public hospital operating suites and recovery areas in Victoria.

**Methods:**

Snapshot audit of procurement data of common medical devices utilised in operating theatres that are available as either reusable or single‐use devices. A list of 49 common medical devices was created, and the usage data were obtained through surveys conducted by remote interviews of procurement or theatre staff between August 2024 and January 2025. Descriptive statistics were used to summarise data. Nine public health networks (i.e., 14 hospitals) were surveyed, comprising 121 operating theatres and 21 endoscopy/procedure rooms.

**Results:**

Eight out of nine (89%) metropolitan public health services in Victoria use mostly single‐use surgical and anaesthetic devices in the operating theatre. Of the 49 surgical and anaesthetic devices surveyed, 46/49 (94%) had a reusable alternative available at a minimum of one of the nine health services surveyed. Five of 16 (31%) anaesthetic devices, 15/27 (56%) surgical devices, and 2/7 (29%) recovery room devices had a reusable alternative available. The average hospital adoption of surveyed reusable devices across all sites was 22/49 (45%).

**Conclusions:**

Most (8/9) public health services in Melbourne surveyed utilise mostly single‐use devices in perioperative settings. This audit provides a snapshot where reusable alternatives are available to influence future procurement. As the Victorian healthcare system rapidly transitions to renewable energy sources of electricity, there are considerable procurement opportunities to reduce scope three greenhouse gas emissions from healthcare.

## 1 Background

Climate change is widely recognised as the greatest threat to human health in the 21st century, yet the healthcare sector paradoxically remains a significant contributor to global greenhouse gas emissions [1]. In Australia, healthcare contributes 5%–7% of total national carbon dioxide emissions (CO_2_e) [2]. A considerable proportion stems from the perioperative environment owing to the large consumption of energy, production of waste, and emissions associated with the supply chain [3].

Recommendations for the decarbonisation of Australia's healthcare systems include transitioning towards reusable devices [4, 5, 6]. The environmental impact of single‐use compared to reusable medical devices is greatly influenced by the energy source of the healthcare facility, with nonrenewable energy sources contributing to higher emissions compared to facilities powered by renewable energy [7]. In countries with a greater proportion of their electricity supplied by renewable sources (e.g., the United Kingdom/Europe), the transition to reusable anaesthetic devices can result in a large (e.g., 84%) reduction in CO_2_ emissions [7]. As the Australian energy sector transitions rapidly towards renewable energy, the environmental advantages of reusable devices augment considerably [8]. In addition to reducing greenhouse gas emissions, the use of reusable devices in the perioperative setting has been associated with financial savings [7].

HealthShare Victoria (HSV) is an independent statutory authority that procures for the Victorian public health system. The 2024–2025 HSV Statement of Priorities supports health services to be more resilient to climate change as part of the Department of Health Strategic Plan 2023–2027 [9]. These goals include supporting health services to estimate Scope Three CO_2_ emissions that is, indirect emissions from the supply chain, such as procured medical devices [9]. Approximately 70% of carbon dioxide equivalent (CO_2_e) emissions from the Australian healthcare sector are attributable to Scope 3 sources. Targeting procurement practises therefore represents a significant opportunity to reduce emissions [4, 10].

We aimed to understand the current adoption of reusable devices across Victorian public health services. We sought to interview healthcare staff within networks associated with the University of Melbourne to identify the adoption of commonly used single‐use and reusable anaesthetic, surgical and recovery room devices.

## 2 Methods

Ethics approval for this audit was granted by Austin Health (Heidelberg VIC, Australia) Ethics committee HREC/108878/Austin‐2024.

A list of common medical devices was developed based on knowledge that: (i) a reusable alternative had been used within a local hospital (authors J.D., F.M.), (ii) the reusable and single‐use devices had previously been compared using life‐cycle assessments (J.D., F.M.) [11] and (iii) devices were broadly described to avoid doubling up or incorrect identification (EH, AD, JD, FM). This list of 49 items was appraised by all four authors and agreed upon (Table 1).

**TABLE 1:** Devices examined (anaesthesia, surgical, recovery room).

Anaesthesia devices (*n* = 16)	Surgical devices (*n* = 26)	Recovery room devices (*n* = 7)
**Airway devices** Anaesthesia circuit tubing Bag‐mask resuscitator facemask (all sizes) Bronchoscope (all sizes) Laryngeal mask airway (all sizes) Direct laryngoscope (Macintosh) blade (all sizes) Video laryngoscope blade (all sizes) **Procedural devices** Artery forceps Central line access kit (towels, metal instruments, kidney dishes, pots) Needle holder Regional anaesthesia tray/kit Scissors Medication tray **Textiles** Sterile drape (spinal procedures/central line insertions) Sterile gown **Noninterventional devices** Blood pressure cuff (all sizes) Venous tourniquet	**Textiles** Sterile drapes Sterile gowns Theatre caps **Surgical instruments** Equipment trays (all sizes) Forceps (individual) Laparoscopy instruments (majority) Needle holder (individual) Scissors (individual, outside of pack) **Associated devices** Bowls (all sizes) Gallipots (all sizes) Instrument quiver Jugs (all sizes) Kidney dishes (all sizes) Sharps tray **Endoscopes** Colonoscope Cystoscope Duodenoscope Gastroscope **Packs** Catheter kit (IDC) Minor suture/wound kit/dressings pack **Theatre technician devices** Arterial tourniquet Hover mat Nonslip pads and mats for patients (on surgical table) (gel pads) Non‐slip strapping (on surgical table) Slide sheets	Plastic bowls Denture cups Drinking cups Jug Kidney dish Medication trays Pill cups

A script of four standard questions (Table 2) was generated in relation to the 49 pieces of devices (Table 1). Contact with appropriate personnel to complete the interview was determined via initial phone calls to nurse unit managers or procurement offices at the University of Melbourne affiliated teaching hospitals (E.H., A.D.). This study focused on health services affiliated with the University of Melbourne to ensure feasibility of data collection, including established academic relationships with the authors.

**TABLE 2:** Question list used in interviews.

Survey questions
Is the following device at your institution single‐use, reusable or both? If both devices are available, is the single‐use or reusable alternative more commonly used? If the device is reusable, is it disinfected or sterilised within the Central Sterilisation Services Department (CSSD) at your hospital or an alternative site?

Invitations to interviews were then sent via email correspondence to the appropriate procurement or theatre staff with the necessary expert knowledge of the devices included at each respective health service. Data were collected via remote interviews (either via telephone or video conference) with these key stakeholders. These interviews were conducted by E.H. and A.D. and were not recorded. If all questions were unable to be completed in a single interview, or if the participant was unable to commence the interview, follow‐up correspondence to complete the survey was conducted via e‐mail. One participant was recruited from each of the nine health services.

Results from interviews were tabulated in a secure Microsoft Excel spreadsheet and collated. Preferred use was determined from participants' responses to their knowledge if reusable or single‐use was more commonly used at their hospital.

The devices were categorised into Anaesthesia, Surgery, and Recovery (Table 1). The adoption of reusable alternatives for each category and subcategory across all hospitals surveyed was calculated and tallied.

## 3 Results

This multicentre, cross‐sectional survey of procurement data across nine public metropolitan health services affiliated with the University of Melbourne was conducted between August 2024 and January 2025.

All nine (out of nine) health services invited to participate in this study responded, with results from 14 individual hospitals consisting of a total of 121 individual operating theatres and 21 endoscopy or procedure rooms. All participants were knowledge experts on medical devices used in their areas (e.g., procurement managers or theatre staff). In this study, 44% (4/9) respondents were theatre staff such as theatre nurse unit managers or associate theatre nurse unit managers and 55% (5/9) were procurement managers.

### 3.1 General Trends

Of the 49 devices surveyed, 46/49 (94%) had at least one reusable option adopted at one or more of the hospitals surveyed. The overall adoption of reusable alternatives per hospital by number of devices across all sites was 22/49 (45%).

Table 3 summarises the adoption of reusable alternatives for items across the 14 hospitals surveyed and included. Table 4 depicts average adopted reusable alternatives across all health services surveyed (the 14 hospitals surveyed and included belonged to nine health services).

**TABLE 3:** Summary of products surveyed and the number of hospitals (out of 14) reporting adoption of a reusable alternative of the specified device.

Device	Number of hospitals (out of 14) where a reusable device was available
Anaesthesia devices (16 devices)	
Anaesthesia circuit tubing	8
Macintosh blade (all sizes)	10
Video laryngoscope blade (all sizes)	7
Laryngeal mask airway (all sizes)	3
Bag‐mask ventilation facemask (all sizes)	3
Regional anaesthesia tray/kit	3
Central line access/insertion kit	1
Artery forceps	7
Needle holder	7
Scissors	6
Bronchoscope (all sizes)	9
Sterile gown	5
Sterile drape (spinal procedures/central line insertions)	4
Medication tray	6
Venous tourniquet	7
Blood pressure cuff (all sizes)	12
Surgical devices (26 devices)	
Sterile drapes	6
Sterile gowns	6
Towel (huck)	6
Theatre caps	5
Sharps tray	2
Instrument quiver	8
Kidney dishes (all sizes)	6
Bowls (all sizes)	6
Gallipots (all sizes)	4
Jugs (all sizes)	8
Surgical equipment trays (all sizes)	10
Scissors (individual, outside of pack)	12
Forceps (individual)	12
Needle holder (individual)	11
Laparoscopy instruments (majority)	11
Catheter kit (IDC)	0
Minor suture/wound kit/dressings pack	4
Gastroscope	12
Colonoscope	11
Duodenoscope	12
Cystoscope	10
Arterial tourniquet	5
Hover mat	8
Slide sheets	11
Non‐slip pads and mats for patients (on surgical table)	11
Non‐slip strapping (on surgical table)	9
Recovery (seven devices)	
Pill cups	0
Kidney dish	7
Medication trays	6
Bowl	4
Jug	6
Drinking cups	4
Denture cups	0

*Note*: Cell shading represents a 14‐colour heatmap gradient, where increasing green intensity indicates greater adoption of reusable alternatives by hospitals, and increasing red intensity indicates lower adoption (0 = most intensely red; 14 = most intensely green).

**TABLE 4:** Health services that have adopted reusable devices or single‐use devices (where there is a choice available).

Item	HS1	HS2	HS3	HS4	HS5	HS6	HS7	HS8	HS9
Anaesthesia equipment (*N* = 16)									
Airway devices									
Anaesthesia circuit tubing									
Macintosh blade (all sizes)									
Video laryngoscope blades (all sizes)									
Laryngeal mask airways (all sizes)									
Bag‐mask ventilation facemask (all sizes)									
Bronchoscope (all sizes)									
Procedural devices									
Artery forceps									
Scissors									
Needle holder									
Medication tray									
Central line access kit									
Regional anaesthesia kit									
Textiles									
Sterile gown (spinal/sterile procedures)									
Sterile drape (spinal/sterile procedures)									
Non‐interventional devices									
Blood pressure cuff (all sizes)									
Venous tourniquet									
Surgical equipment (*N* = 26)									
Textiles									
Sterile drapes									
Sterile gowns									
Theatre caps									
Huck towel									
Surgical instruments									
Surgical equipment trays (all sizes)									
Forceps (individual)									
Laparoscopy instruments (majority)									
Needle holder (individual)									
Scissors (individual, outside of pack)									
Hollow ware									
Bowls (all sizes)									
Gallipots (all sizes)									
Instrument quivers									
Jugs (all sizes)									
Kidney dishes (all sizes)									
Sharps tray									
Endoscopes									
Colonoscope									
Cystoscope									
Duodenoscope									
Gastroscope									
Packs									
Catheter kit (IDC)									
Minor suture/wound kit/dressing pack									
Theatre technician devices									
Arterial tourniquet									
Hover mat									
Non‐slip pads and mats for patients									
Nonslip strapping									
Slide sheets									
Recovery room equipment (*N* = 7)									
Kidney dish									
Medication tray									
Bowl									
Jug									
Pill cups									
Denture cups									
Drinking cups									

*Colour Key*: 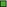, reusable; 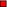, single use; 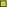, both (more reusable); 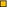, both (more single use); 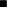, no data available.

*Note*: Devices are listed alongside the health services that have adopted reusable devices, indicated by green colouring and those that have adopted a single‐use device. Services where both devices in reusable or single‐use form are available are chartreuse where the reusable option is preferred by clinicians, and orange where the single‐use option is preferred. Services where the item is unavailable at all are indicated in black.

Abbreviation: HS = health service.

### 3.2 Anaesthesia

The most common reusable anaesthetic devices were blood pressure cuffs, laryngoscope blades and bronchoscopes.

The most common single‐use anaesthetic devices used in anaesthetics included central line insertion kits, bag valve ventilation masks, laryngeal mask airways and regional anaesthesia kits. Metallic instruments used in anaesthesia were mostly single‐use.

### 3.3 Surgical

The most commonly reusable devices surveyed from the surgical category were metallic instruments (artery forceps, needle holders, etc.). Comparable metal instruments used in anaesthesia were predominantly single‐use, whereas identical instruments within the surgical category were routinely reprocessed and reused via CSSD.

The most common single‐use items from this category were sharps trays, gallipots, catheter packs, surgical gowns and drapes.

Two health services (five hospitals) had access to reusable sterile surgical gowns, and three health services (five hospitals) had access to reusable sterile drapes. Of these hospitals that had reusable textiles, only one health service predominantly used the reusable option when both options were available. All other health services used solely single‐use textiles, even when a reusable option was available.

### 3.4 Recovery Room

The most common items for recovery were kidney dishes followed by medication trays and jugs. Most common single‐use items in the recovery space were pill cups, drinking cups, and denture cups. All hospitals used single‐use pill cups and denture cups.

## 4 Discussion

We provide a multi‐centre assessment of reusable and single‐use perioperative devices in Australian public healthcare. Despite evidence identifying environmental and financial benefits to transition towards reusable devices, only one health service out of the nine (i.e., three hospitals out of 14 surveyed) was utilising a majority of single‐use items.

This study was novel in design. Despite the financial and environmental benefits of reusable devices, only anecdotal evidence has existed regarding the availability of reusable devices in Victorian public hospitals. A key limitation of this study was the reliance on verbal reports from procurement staff, without independent verification of the actual usage or availability of the reported items. Due to confidentiality constraints surrounding procurement contracts, we were unable to access objective data on the quantities of reusable and single‐use devices procured. Another limitation is the absence of data on the mass of surveyed items. A key concern we identified was the relative scarcity of reusable surgical textiles. Surgical drapes and gowns contribute substantially to overall waste volume and likely have a greater environmental impact compared to smaller items. When considering the number of gowns and drapes consumed per theatre case, the volume of waste and associated Scope 3 emissions is significant. Surgical drapes and gowns contribute substantially to overall waste volume and likely have a greater environmental impact compared to smaller devices. In this study, six hospitals reported access to either reusable gowns or reusable drapes, but only one health service routinely used these reusable textiles. Despite an average of 45% of surveyed devices in Victorian hospitals being reusable, disposable surgical gowns and drapes have a likely disproportionate contribution to waste due to their mass.

The Royal Australasian College of Surgeons supports the use of reusable surgical gowns, and through its statement has encouraged a safe and structured transition towards reusable gowns [12]. As such, it would be useful to explore the mass and volume of gowns and drapes utilised at these health services, and their associated life cycle assessments to compare the carbon footprints associated with operating theatres relying on mostly reusable gowns and drapes and those using mostly single‐use options. A further limitation was the noninclusion of pulse oximeters in our list of surveyed devices. Initially, this was because anecdotally within the local Melbourne public health service context, most pulse oximeters were presumed to be reusable based on the authors' experiences. However, in other parts of Australia, single‐use pulse oximeters remain in use. Given the monitoring standards recommended for general anaesthesia and sedation [13], a transition to reusable pulse oximetry systems could yield substantial cost savings for health services.

There is limited literature examining international practises regarding the availability and adoption of reusable surgical and anaesthetic devices [14]. Reusable laparoscopic and endoscopic instruments appear to be commonly utilised, potentially driven by considerations of cost reduction [15, 16]. There is probably a greater use of disposable surgical devices in high‐income countries [17]. This trend may be partly explained by commonly cited barriers to the adoption of reusable options, including concerns about infection prevention, despite existing evidence demonstrating noninferiority of reusable items and no associated increase in infection risk when compared to single‐use alternatives [18, 19, 20, 21]. Feasibility in switching to reusable options can be described by the capability, opportunity and motivational model of behavioural change (COM‐B) [21, 22]. Table 4 outlines current capabilities of Victorian healthcare and HSV in the procurement of reusable devices listed and highlights opportunities for Victorian health services who have not yet adopted reusable alternatives to switch to reusable procurement options available in the state. Whilst we did not survey staff regarding perceived barriers in the transition to reusable devices in this study, a recent Australian qualitative study [21] identified implementation challenges, including the need to address misconceptions around the safety of reusable devices, secure funding to support the additional workload associated with disinfection and sterilisation processes, and revise procurement pathways. Additional factors that are more complex and difficult to address acutely include individual surgeon preferences for equipment and marketing towards individual hospitals or practitioners [21]. Similar barriers have been reported in European, US and UK studies [23, 24, 25].

Procurement of surgical and anaesthetics equipment has traditionally been driven by cost. Prior to 2000, Australian public hospitals were reprocessing equipment intended for single use only, for cost saving purposes [26]. In the 1990s to 2000s we saw an increase in disposable equipment due to infection concerns; however, evidence now supports that reusable devices are not a risk factor for surgical site infections [27]. Now, the rationale for health services in procuring reusable versus single‐use devices can be attributed to their cost; both fiscal and environmental. Whilst there are no Australian studies comparing cost of reusable and single‐use surgical equipment, transitioning to reusable anaesthetics equipment saves approximately 5000 AUD per operating suite or 100 000 AUD per annum at a large metropolitan health service in Victoria [7]. Switching to reusable equipment is likely to result in significant financial savings for health services.

As our electricity mix transitions to renewable, the environmental viability of reprocessing and sterilising reusables improves [7, 28]. Victorian public health services have traditionally operated on electricity sourced primarily from brown coal [29]. As of 1 July 2025, public hospitals in Victoria have transitioned to predominantly renewable electricity supply through the State Electricity Commission [30]. This considerably decreases environmental costs associated with re‐processing and sterilisation of reusable devices. ACT, QLD and TAS have already achieved 100% renewable supply for public healthcare, with similar commitments to achieve net zero from other Australian states and territories [29]. Scope three emissions make up a significant proportion of total Australian healthcare emissions (68%) [2], and as Australian healthcare transitions towards a fully renewable energy supply, now is an opportune time to prioritise reusable medical devices to maximise both environmental and economic benefits. This study has identified key areas, particularly medical textiles and plastic holloware, for further exploration in reducing scope three emissions within Victoria's healthcare system, aligning with the goals outlined in the National Health and Climate Strategy [4] and the aligning with efforts of the Australian Commission on Safety and Quality in Healthcare to introduce sustainability as an integral component of healthcare facility accreditation and quality assurance [31].

## 5 Conclusions

We surveyed 49 commonly used items that had reusable and single‐use variants available from surgery, anaesthesia, and the postanaesthesia recovery room from 14 hospitals (nine health services) in Victoria. Of the 49 products surveyed, 46/49 (94%) had a reusable option available within at least one of the hospital sites surveyed. The average amount of reusable alternatives per hospital across all sites was 22/49 (45%). There is significant scope to develop a comprehensive inventory of reusable items in Australian healthcare. The successful adoption of predominantly reusable items in the operating theatre by one major Victorian health service demonstrates the feasibility of broader implementation across the sector. As our healthcare system transitions to renewable energy sources, there are significant opportunities to reduce the operating room's carbon footprint, whilst maintaining excellent peri‐operative care.

## Conflicts of Interest

Forbes McGain receives royalties for the sale of the McMonty personal protective hood for patients with respiratory infections.

## Data Availability

The data that support the findings of this study are available from the corresponding author upon reasonable request.
